# Knowledge of tuberculosis management using directly observed treatment short course therapy among final year medical students in South Western Nigeria

**DOI:** 10.11604/pamj.2014.18.32.3553

**Published:** 2014-05-08

**Authors:** Olarewaju Sunday Olakunle, Olanrewaju Oladimeji, Adebimpe Wasiu Olalekan, Adenike Olugbenga-Bello, Callistus Akinleye, Olarewaju Abiodun Oluwatoyin

**Affiliations:** 1 Department of Community Medicine, Ladoke Akintola University of Technology, Ogbomoso, Oyo State; 2 Clinical Sciences Department, Management Sciences for Health, Abuja, Nigeria; 3 Department of Community Medicine, College of Health Sciences, Osun State University, Osogbo Nigeria; 4 Department of Community Medicine, Ladoke Akintola University of Technology Teaching Hospital, Osogbo, Nigeria; 5 Department of Medical Microbiology and Parasitology, Ladoke Akintola University of Technology, Ogbomoso, Oyo State

**Keywords:** Tuberculosis, Directly observed treatment short course therapy (DOTS)

## Abstract

**Introduction:**

Equipping medical graduates with the competence to manage tuberculosis is not just imperative but also urgent as the diseases have been consistently listed as one of the major causes of morbidity and mortality in Nigeria. However, there were no baseline studies done on knowledge of final year medical students on various aspects of TB diagnosis and management under directly observed treatment short course therapy (DOTS) which forms the basis of this study.

**Methods:**

A total of 241 final year medical students from three medical colleges in Nigeria were interviewed. The questions assessed their knowledge about various modes of transmission, symptoms and management of tuberculosis under DOTS.

**Results:**

More than half of the respondents (i.e. 69%) had poor knowledge on TB disease. Only 33.6% mentioned sputum smear as the best tool of diagnosing TB according to guideline. Poor knowledge was also exhibited when asked of various categories under DOTS treatment regimen, as 46.1% correctly mentioned cat 1 and 2. Minority 18.7% and 6.7% had complete knowledge of 6 months duration for new TB cases and 8 months for re-treatment cases respectively. Less than one tenth, i.e. 4.6% and 2.9% could correctly defined what is called a new TB case and re-treatment cases according to standard guideline.

**Conclusion:**

The study reveals gross inadequacies in TB knowledge and management practices among Nigerian final year medical students. There is urgent need for incorporation of National TB guideline into existing undergraduate medical education curriculum as well as students rotation through activities in DOTS clinic.

## Introduction

Tuberculosis (TB) has re-emerged as a major global public health concern since the mid-1980s. Globally, Tuberculosis accounted for 1.2 - 1.5 million deaths (including mortality due to Tuberculosis as well as TB and HIV co-infection), 85% of this occurring in developing countries and 26% in Africa [1]. Thus, TB ranks as the second highest cause of adult mortality after HIV in the world. It also tends to affect men more than women, mostly among the economically productive age group. [1] Factors contributing to the re-emergence include pervasive poverty and lack of good governance, the HIV/AIDS epidemic poor public health services, rapid population growth and rapid urbanization [2].

TB is a highly contagious and fatal disease of public health concern particularly in low income countries. [3] It remains a major cause of high morbidity and mortality in these countries despite considerable decline in prevalence in the developed world [4]. Unfortunately, it affects mainly the economically productive age group despite the availability of cure [5]. The disease spreads through air by droplet nuclei and the micro-organisms enter the body through lung inhalation. So, only people with pulmonary TB are infectious. This form of disease is the most frequent, [3] occurring in more than 80% of cases. The extra-pulmonary TB are almost never infectious, unless with concomitant pulmonary TB [3]. However, TB could actually affect any part of the body. Most infected people (80-90%) will never become ill with TB unless with seriously compromised immunity. Nevertheless, each active TB case will infect on average between 10 and 15 people every year [1, 5].

In Nigeria, there were 33,000 deaths resulting from TB disease in year 2009. [1] Despite the availability of proven interventions such as the use of anti-TB drugs reported to produce a cure rate of up to 87%, there are differing trends in the incidence of re-treatment and new MDR cases. Likewise, there is a strong political commitment to combat TB and this has led to the establishment of National Tuberculosis and Leprosy Control Programme (NTBLCP), an arm of the Federal Ministry of health given the mandate to control TB and leprosy in Nigeria. The vision of the Programme is “Nigeria free of TB” while its goal is to reduce TB to a level at which it is no longer a public health problem. In line with this vision, the NTBLCP adopted the WHO recommended DOTS strategy in 1993 and the Stop TB strategy 2006 and has since then scaled-up implementation to all the 36states and FCT with significant improvement in DOTS expansion from 40% in 2006 to 63% in 2010 (1 DOTS centre/25,000 population) to achieve 70% case detection and 85% cure rate. [6] There has been a steady increase in total number of all forms of TB cases notified from 90,311 in 2008; 90,447 in 2010 to 93,050 in 2011 (the latter representing a CNR of 58/100,000 pop for all forms of TB) still below the 70% case detection target [7].

Medical schools are one of the important portals for management of patients with TB. DOTS centers have also been established in these colleges to increase access to TB treatment. Medical schools play an important role not only in the building of medical expertise but also in the socialization of future physicians. Societies expect these institutions to train students to competently and holistically handle common health problems. To widen access and improving quality of TB services as well as for giving hands on training to students, involvement of medical colleges is paramount. Knowledge of tuberculosis is assimilated in parts over all the years in the medical college. A TB clinic posting exposes the medical student to the practical aspects of Tuberculosis treatment, giving them an insight in to the day to day working of a DOTS clinic. Medical colleges play a central role in training and shaping the attitudes of the future generations of medical practitioners.

However, there is a dearth of data regarding the level of knowledge about TB and DOTS among medical students who are the budding doctors and can make an impact on TB control. Previous studies conducted in Nigeria and other countries worldwide focuses on the knowledge of TB and its management among practicing doctors, both in private or public sector showing considerable variation in prevention, evaluation and treatment strategies, indicates less than optimal performance and highlight the need for further education and training in issues relating to tuberculosis among physicians [8–12].

Physicians in the future need to be aware of the epidemiology, determinants, screening, and management of re-emerging infections such as tuberculosis. Increased exposure and education in both academic and clinical settings is crucial if medical students are to become competent in this arena. In view of the above background, this study was conducted with objective of assessing knowledge of the final year medical students in three medical colleges in Nigeria, about various aspects of diagnosis and management of TB under Directly observed treatment short course therapy (DOTS).

## Methods

This study was carried out in Southwestern Nigeria. Government of Nigeria adopted the WHO recommended DOTS strategy as the national modality for the treatment of Tuberculosis, and the strategy had been effective in Nigeria like in many other parts of the world. However, most TB programmes are donor driven, though the federal Government through the NTBLCP coordinates TB response efforts in the country. There are 8 medical schools in the Southwestern region, 4 owned by Federal and 4 owned by states governments. Lectures on DOTs and rotation through PHCs and DOTs centers are usually incorporated into the medical school curriculum which final year medical students would have passed through before certified as a medical doctor

This is a descriptive cross sectional study among medical students in Southwestern Nigeria. All medical students in their final year constitute the target population. Eligible participants were registered final year MBBS students in selected medical schools. Sample size was estimated using the Leslie's Fischer's formular for single proportion using a prevalence of 16%. The minimum calculated sample size of 206 was increased to 242 to take care of non response.

Sampling was done in the multistage fashion. Three out of 8 medical schools in Southwestern Nigeria were selected using simple random sampling employing simple balloting. In stage 2 and for a medical school, 2 out of 4 groups of final year medical students on clinical rotation were also randomly selected using simple balloting. Questionnaires were equally allocated in each sampling stage. In stage three, a list of medical students per rotation group was made, and a systematic sampling method of 1 in 3 names on the list was made to reach the respondents for this study.

Research instruments were semi structured self administered pre tested questionnaires administered by trained lecturer assistant from each of the selected medical schools. Study variables include their knowledge, perception and practice of DOTs regimen including diagnosis and management and treatment of tuberculosis. The questionnaires had multiple choice questions, and also single or multiple responses of possible options that were correct. The subjects had a choice of not answering any question they did not know. Data were collected over a period of 3 months after making a total of 6 visits to the medical schools.

Ethical approval to conduct the study was obtained from LTH ethical review committee while a written consent was obtained from each respondent. A total of 241 finalists were interviewed. Data collected was analyzed using SPSS statistical package after data cleaning, and ensuring data validity through random checks and double entry of data. Frequency data were generated. Both bi and multivariate logistic regression were done in addition to Chi squared testing to demonstrate association between variables of interest. P value was set at less than or equal to 0.05 for all inferential statistics having to do with significance tests.

## Results

All two hundred and forty one respondents returned useful and completely filled questionnaires. The respondent's age ranged between 20 - 49 years with a mean age 26 + 3 years and a modal age group 25 - 29 years. There were more males 147 (60.7%) than female 95 (39.3%). Majority of the respondents were Christians 189 (78.1%) while the remaining 52 (21.9%) were Moslems (Table 1).

**Table 1:** Socio-demographic status of respondents

Variable (N = 241)	Frequency	Percentage
**Sex**		
Male	147	60.7
Female	95	39.3
**Marital status**		
Single	204	84.0
Married	36	14.9
Separated	2	0.8
**Religion**		
Christian	189	78.1
Moslem	52	21.9
**Ethnicity**		
Yoruba	179	74.0
Hausa	10	4.0
Ibo	53	22.0
**Age group**		
20 -24	70	28.9
25- 29	150	62.0
30-34	18	7.0
35-39	3	1.2
40 -44	1	0.4

Likewise in terms of TB diagnosis according to National guideline, 87 (33.6%), 85 (35.3%) and 73 (30.3%) mentioned sputum smear, chest x-ray and sputum culture respectively. In addition, only 29 (11.9%) was able to mention three methods used for diagnosis tuberculosis i.e. chest x-ray, sputum smear and sputum culture (Table 2).

**Table 2:** Knowledge on TB diagnosis and follow up by National Guideline

Variable (Multiple responses allowed)	Frequency	Percentage
Sputum smear	87	33.6
Chest x-ray	85	35.2
Sputum culture	73	30.3
Using three methods (Sputum smear, chest x-ray and sputum culture)	29	11.9
Using two methods ( Sputum smear and chest x-ray)	29	11.9
Using one method (Sputum smear only)	88	36.4

The correct classification of patients into Cat 1 and Cat 11 was done by 111 (46.1%) of respondents while only 83 (34.5%), 16 (6.7%), 9 (3.7%) were able to identify correctly regimen duration for new tb, re-treatment and tb treatment among children. However, only 4 (1.7%), 7 (2.9%) were able to define correctly new tb and re-treatment tb cases (Table 3).

**Table 3:** Knowledge of respondents on TB classification and treatment by National Guideline

Variable	Frequency	Percentage
Cat 1 and Cat 11	111	46.1
6 months regimen for new cases	45	18.7
8 months regimen for new cases	38	15.8
8 months regimen for re-treatment cases	16	6.7
6 months regimen for children	9	3.7
Cured definition	4	1.7
New TB case definition	11	4.6
Re-treatment case definition	7	2.9

## Discussion

The study revealed gross inadequacies in the knowledge of tuberculosis management according to DOTS regimen among final year medical students in South Western, Nigeria. Less than half of the respondents mentioned sputum smear, chest X-ray and sputum culture as means of diagnosing tb according to National guideline. A very similar finding was observed among medical interns in Turkey with 28.8% but higher findings recorded among interns in ido-ekiti, South Western Nigeria and Belgore where ZN staining for AFB was identified as the best diagnostic procedure/technique for PTB by 74 (62.7%) and 71.1% respectively [13–15].

Likewise, it is worrisome to know that only 34.5% and 6.7% were able to identify correctly regimen duration for new and re-treatment tuberculosis cases according to standard guidelines. This low level could be the result of deficiency in TB education in most Nigerian medical schools and affiliated teaching hospitals. This is made much worst by absence of effective DOTS centers in many tertiary centers including the teaching hospitals. However, since National Tuberculosis and Leprosy Control Program (NTBLCP) is already in place, though yet to achieve its global targets of 70% case detection despite adoption of DOTS strategy in the early 90s, there is need for additional support by effective and well trained medical practitioners towards achieving the target. The onus lies on the medical colleges and the curriculum to produce well trained and skilled medical practitioners. The knowledge level of graduating doctors and their attitudes may influence national TB control programs.

As far as the rank of Nigeria among the 22 high burden countries for TB, only 1.7% and 2.9% were able to define correctly new tb and re-treatment tb cases. Such low level was observed among final year medical students in tertiary level health facility in India where 16% 5th year medical students were able to classify patients according to drug regimen and category [16]. Knowledge about the terms as cured was also not satisfactory as only 1.7% correctly defined cure. Our result was lower when compared to a similar study among medical finalist in India where 30% of students mentioned correct definitions [16].

There is an urgent need for massive increase in awareness of DOTS among medical students. First, federal government must enforce the establishment of strict and dedicated DOTS clinic in all tertiary hospitals. Second, medical students must rotate through DOTS clinic and practically participate in all its activities, including performance of ZN staining for sputum smear microscopy. The revision of existing medical education curriculum in Nigeria should focus on incorporation of national TB guidelines into TB teachings in schools. The appropriate authority should ensure the circulation and availability of TB guidelines to every practicing medical doctor in the country. This will encourage medical practitioners to inculcate diagnostic and prescription practices that are in accordance with the national TB guidelines [17].

## Conclusion

Tuberculosis being the major public health problem in Nigeria, needs a higher priority in the medical curriculum. The knowledge level of final year medical students in the present study was found to be poor for the various aspects of tuberculosis management under DOTS programme. This study highlights the inadequate and incomplete knowledge of medical undergraduates regarding TB treatment using DOTS, and emphasizes the need for more regular training sessions along with strict supervision of trainees. This study thus concludes that TB/DOTS clinic posting and training should be made mandatory for all the medical students to increase their knowledge and skills for effective management of patients with tuberculosis and thereby in the long run preventing the further rise of MDR and XDR TB cases. As long as TB continues to plague the country, empowering future physicians with competent knowledge of TB and DOTS remains a most viable solution. Based on the experience, we suggest the following: full integration of the TB control-DOTS curriculum across all levels and medical schools in Nigeria; faculty orientation and training on implementing the curriculum in their classes; access to instructional materials on TB control and DOTS; administrative support for the full implementation of the curriculum.
